# Real-world association of adherence with outcomes and economic burden in patients with tuberculosis from South Korea claims data

**DOI:** 10.3389/fphar.2022.918344

**Published:** 2022-08-16

**Authors:** Sun-Hong Kwon, Jin Hyun Nam, Hye-Lin Kim, Hae-Young Park, Jin-Won Kwon

**Affiliations:** ^1^ School of Pharmacy, Sungkyunkwan University, Suwon, South Korea; ^2^ Division of Big Data Science, Korea University Sejong Campus, Sejong, South Korea; ^3^ College of Pharmacy, Sahmyook University, Seoul, South Korea; ^4^ BK21 FOUR Community-Based Intelligent Novel Drug Discovery Education Unit, College of Pharmacy and Research Institute of Pharmaceutical Sciences, Kyungpook National University, Daegu, South Korea

**Keywords:** adherence, tuberculosis, cost, treatment outcome, re-treatment

## Abstract

**Objectives:** We analyzed tuberculosis (TB)-related costs according to treatment adherence, as well as the association between treatment adherence, treatment outcomes, and costs related to drug-susceptible TB in South Korea.

**Methods:** Patients who had newly treated TB in South Korea between 2006 and 2015 were selected from nationwide sample claims data and categorized into adherent and non-adherent groups using the proportion of days TB drugs covered. Patients were followed-up from the initiation of TB treatment. The mean five-year cumulative costs per patient were estimated according to adherence. Moreover, we evaluated the relative ratios to identify cost drivers such as adherence, treatment outcomes, and baseline characteristics using generalized linear models. Four treatment outcomes were included: treatment completion, loss to follow-up, death, and the initiation of multidrug-resistant TB treatment.

**Results:** Out of the 3,799 new patients with TB, 2,662 were adherent, and 1,137 were non-adherent. Five years after initiating TB treatment, the mean TB-related costs were USD 2,270 and USD 2,694 in the adherent and non-adherent groups, respectively. The TB-related monthly cost per patient was also lower in the adherent than in the non-adherent (relative ratio = 0.89, 95% CI 0.92–0.98), while patients who were lost to follow-up spent more on TB-related costs (2.52, 2.24–2.83) compared to those who completed the treatment.

**Conclusion:** Non-adherent patients with TB spend more on treatment costs while they have poorer outcomes compared to adherent patients with TB. Improving patient adherence may lead to effective treatment outcomes and reduce the economic burden of TB. Policymakers and providers should consider commitment programs to improve patient’s adherence.

## Introduction

The ‘End TB Strategy,’ published by the World Health Organization (WHO) in 2015, aims to reduce the number of tuberculosis (TB) deaths by 95%, incidence by 90%, and related catastrophic expenses to 0% by 2035 (Uplekar et al., 2015; WHO, 2015). In 2019, approximately 10.0 million (8.9–11.0 million) people were infected with TB worldwide, although the incidence rate has been declining gradually in recent years. The economic burden of TB remains high and sometimes catastrophic, even in Europe (Diel et al., 2014; Tanimura et al., 2014). Additionally, TB management and control strategies might likely become relatively vulnerable and insufficient because of the coronavirus disease 2019 pandemic that began in the second half of 2019 (Wang et al., 2021; Yang et al., 2021). Isolation, social distancing, and collapse of the medical system have led to reduced access of patients with TB to medical facilities. The World Health Organization predicts that 6.3 million additional cases of TB will occur by 2025 (WHO, 2020).

Currently, TB treatment requires long-term administration of multiple drugs. The treatment periods for drug-susceptible TB (DS-TB) and multidrug-resistant TB (MDR-TB) are 6 and 6–20 months, respectively. Long term treatment of TB is a factor that makes treatment success difficulty. According to the guideline (WHO, 2010), TB program quality is evaluated based on treatment outcome indicators and impact indicators. The treatment outcome indicators consist of cure, treatment success, and default; impact indicators include new TB incidence and mortality (WHO, 2010). Among them, previous cohort studies focused on successful treatments and loss to follow-up (LTFU) (Holden et al., 2020; Soedarsono et al., 2021). Negative attitude towards treatment, limitation of family/social support, dissatisfaction with health services, and economic burden may increase LTFU and non-adherence (Kardas et al., 2013; Soedarsono et al., 2021), which leads to high mortality, spreading of MDR-TB with wasting health resources (Heemanshu and Satwanti, 2016).

Non-adherence to treatment is main challenge not only for clinicians when managing infectious diseases but also for the governments when making the public health policies as a strategy to end TB. Adherence is affected by both the duration and complexity of drug regimens (WHO, 2020). Although medication adherence critically affects the success of TB treatment, many patients with TB (16–49%) do not comply with treatment regimens (Horsburgh et al., 2015). Various global efforts have been made to improve adherence to TB treatment. Directly observed therapy and public-private mix programs have been suggested to maximize adherence and require social and financial support (Lei et al., 2015; Alipanah et al., 2018). Many studies have demonstrated the contribution of public-private mix and directly observed therapy to TB treatment success, and numerous countries have adopted these programs as core strategies to increase adherence to TB management (Slevin et al., 2014). However, the economic impact of adherence has only been evaluated in two studies to date (Lei et al., 2016; Budgell et al., 2018). A study of children and adolescents revealed higher treatment costs for patients whose outcomes were cure or treatment completion than for those who were LTFU or showed treatment failure. Another study observed a higher risk of non-adherence with higher monthly treatment costs in 482 new Chinese patients with TB (Lei et al., 2016; Budgell et al., 2018; Chimeh et al., 2020). However, measurement of direct medical costs according to adherence and comparison of the medical costs between the adherent and non-adherent groups were not performed in these studies.

Cohort analysis is a good tool to evaluate the effectiveness of the national TB plan and improve the plan (WHO, 2010). Therefore, this study was conducted to provide comprehensive evidence of medical costs based on TB adherence using real-world data from approximately 10 years in a representative Korean population. In addition, the factors affecting adherence to treatment, and costs were investigated including treatment outcomes. Determining the treatment outcomes and magnitude of the economic burden can highlight the importance of continued social efforts aimed at improving TB adherence.

## Materials and methods

### Data source

South Korea requires people to be subject to health insurance by law. As a non-profit agency, the national health insurance service (NHIS) is a payer operating health insurance program, which covers the entire national population (approximately 50 million people). The national sample cohort data (NHIS-NSC) was established from health insurance claims data for health-related research. It was sampled at a 2% sampling rate from the entire population as of 2006. About one million individuals who were sampled were observed from 2002 to 2015. For sampling, 2,142 strata were considered, including sex, age, income level by insurance type, and regional divisions. It contains all patients’ demographic characteristics, healthcare resource utilization, codes for disease diagnosis, dates of death, prescription drugs, and medical costs including copayment and reimbursed cost of medical services covered by NHI. Disease diagnosis codes were identified according to the International Statistical Classification of Diseases and Related Health Problems, 10th revision (ICD-10, 2016). We used NHIS-NSC to identify the target study population and evaluate patient outcomes.

### Study design and target population

This was a retrospective cohort study using nationwide claims data. Patients were included in the study if they were identified as having a prescription for anti-TB treatment for 1 month from July 1, 2006, to December 31, 2012 (index period). Each prescription required at least three combinations of DS-TB drugs: isoniazid, rifampin, ethambutol, pyrazinamide, and rifabutin. All patients were required to have filled their initial prescription during the index period, with the first date identified as the index date, and to have claims from 6 months before the index date (pre-index period) until death or end of the index period after the index date (post-index period). Those patients were not MDR-TB at the index date because they used either isoniazid or rifampicin. The Supplementary Figure S1 (online material) shows the study design, and the timeframe on which assessing the inclusion and exclusion criteria for patients, covariates and outcomes.

Patients were excluded from the cohort to avoid complications from prior treatments or any other conditions based on the following criteria: 1) patients younger than 20 years of age on the index date; 2) patients diagnosed with cancer (ICD-10 codes: C00-C97, D00-D09, and D37-D48) or who had claims for organ transplant (ICD-10 code: Z94; Exempted Calculation of Health Insurance) from January 1, 2006, to December 31, 2015; and 3) patients who had been administered any prescription for anti-TB drugs during the pre-index period, including isoniazid, rifampin, ethambutol, pyrazinamide, or rifabutin.

### Treatment outcomes

We focused on the association between patient adherence to anti-TB treatment and medical costs. Patients were assigned to adherent or non-adherent groups according to the proportion of days covered (PDC), which is a commonly-used, conservative, accurate measure of adherence (Martin et al., 2009; Nau, 2012). It reflects the adherence of patients who are prescribed multiple medications concurrently and should count the days where a person had one or more medications available on a daily basis. PDC in our study was calculated as the number of unique days a drug is available within 6 months after the index date divided by 180 days (6 months). A threshold of 80% was used to assign a patient to the adherent group, which has been widely used in previous studies (Nau, 2012; Forbes et al., 2018).

Medical costs were defined as the total amount of medical services and prescriptions. We also considered specific expenses, such as in-patient care, medication/injection, monitoring, surgery/procedure, consultation, and imaging/radiotherapy. TB-related costs were defined as all costs with claims that included anti-TB treatments, such as isoniazid, rifampin, ethambutol, pyrazinamide, and rifabutin. Medical costs were converted to equivalent USD at an exchange rate of 1,116.95 KRW/USD in 2021.

To determine the treatment characteristics of patients with TB, we considered four treatment outcomes according to guideline and previous studies (WHO, 2010; Holden et al., 2020; Soedarsono et al., 2021): treatment completion, LTFU (treatment discontinuation), initiation of MDR-TB treatment, and death. Since claims data didn’t have patients’ sputum smear or culture status, we couldn’t capture treatment failure. Instead, we focused on LTFU and treatment completion like previous studies (Holden et al., 2020; Soedarsono et al., 2021). Treatment completion and LTFU were distinguished by the gap in time during which there were no claims for TB. Based on the TB treatment guidelines, we considered the case using drugs over 6 months (150 days with a window period of 30 days) as treatment completion. LTFU was defined with a 60-days gap within first 6 months. Also, re-treatment was considered as an impact indicator (incidence of relapsed TB). It was defined when TB treatment was captured after initial treatment completion or LTFU.

### Statistical analysis

In primary analyses, we examined the relationship between adherence and all-cause or TB-related costs. We estimated the cumulative cost by considering a weighted available sample estimator (Zhao and Tian, 2001). The incompleteness of follow-up data is a common problem when estimating medical costs. To address this issue, we applied a weighted available sample estimator to adjust for censored patients. In addition, the cumulative cost was examined using several subcomponents of total medical costs.

We also estimated the medical costs per patient per month for each outcome: treatment completion, treatment discontinuation, MDR-TB treatment initiation, and death. Generalized linear models with the gamma distribution and log links were applied to determine the risk factors associated with total costs and TB-related costs per patient per month. Five dimensions, WHO suggested (WHO, 2003; Kardas et al., 2013), were considered to select covariate for model. Age and sex were included as patient-related factor, type of patients insurance plan as the healthcare system related, CCI and comorbidities as the clinical condition related, and a provider of index claims as the socio-economic. Since more than 90% of patients used the HREZ regimen, we didn’t include therapy-related factors.

The descriptive statistics, mean and standard deviation for continuous variables, and sample size and proportion for categorical variables based on adherence status were used to describe the characteristics of patients: age, sex, treatment regimen, type of insurance, type of provider, comorbidity, and Charlson comorbidity index (CCI). Logistic regression analysis was conducted to estimate the odds ratio (OR) of adherence and 95% confidence interval (CI). Survival analysis with competing risks was performed to examine the cumulative incidence rate of treatment outcomes. All analyses, except for the estimation of cumulative cost, were conducted using SAS Enterprise Guide (version 7.13; SAS Institute, Cary, NC, United States). The cumulative cost was estimated using R software (version 3.3.3; The R Project for Statistical Computing, Vienna, Austria). This study was approved by the Institutional Review Board of Sungkyunkwan University (IRB No. SKKU 2020-10-006).

## Results

### Baseline characteristics and adherence

A total of 3,799 patients with DS-TB were included in this study; of these, 2,662 (70.07%) were categorized into the adherent group and 1,137 (29.93%) into the non-adherent group. The patients were followed-up for a mean of 5.30 years. Table 1 shows the baseline characteristics of patients with DS-TB according to their adherence. The mean age of the patients in the DS-TB group was 50.25 years (SD, 18.81). 3,473 patients (91.42%) were treated with a combination of isoniazid, rifampin, ethambutol, and pyrazinamide (HREZ regimen). The adherent group was younger [48.52 (18.25)] than the non-adherent group [54.28 (19.29)]. The proportion of females was higher in the adherent group (45.95%) than in the non-adherent group (40.46%). The mean CCI was higher in the non-adherent group (1.49) than in the adherent group (1.26).

**TABLE 1:** Baseline characteristics according to adherence.

	Total (*n* = 3,799)	Adherent group (*n* = 2,662)	Non-adherent group (*n* = 1,137)
Age, mean (SD)	50.25 (18.81)	48.52 (18.25)	54.28 (19.49)
Age, n (%)			
20‒39	1,310 (34.48)	1,011 (37.98)	299 (26.30)
40‒59	1,226 (32.27)	851 (31.97)	375 (32.98)
60‒79	992 (26.11)	667 (25.06)	325 (28.58)
≥80	271 (7.13)	133 (5.00)	138 (12.14)
Sex, n (%)			
Female	1,684 (44.33)	1,224 (45.98)	460 (40.46)
Male	2,115 (55.67)	1,438 (54.02)	677 (59.54)
Follow-up period (year), mean (SD)	5.30 (2.21)	5.39 (1.97)	5.10 (2.67)
PDC, mean (SD)	0.8 (0.23)	0.92 (0.06)	0.52 (0.23)
Treatment regimen, n (%)			
INH + RIF + EMB + PZA (HREZ)	3,473 (91.42)	2,472 (92.86)	1,001 (88.04)
INH + RIF + EMB (HRE)	255 (6.71)	148 (5.56)	107 (9.41)
INH + RIF + PZA (HRZ)	50 (1.32)	30 (1.13)	20 (1.76)
INH + EMB + PZA	11 (0.29)	6 (0.23)	5 (0.44)
RIF + EMB + PZA	8 (0.21)	5 (0.19)	3 (0.26)
EMB + PZA + rifabutin	1 (0.03)	1 (0.04)	-
INH + EMB + PZA + rifabutin	1 (0.03)	-	1 (0.09)
Type of insurance, n (%)			
Health insurance	3,499 (92.1)	2505 (94.1)	994 (87.42)
Medical aid	300 (7.90)	157 (5.90)	143 (12.58)
Type of provider at index, n (%)			
Tertiary hospital	1,184 (31.17)	908 (34.11)	276 (24.27)
General hospital	1,690 (44.49)	1,172 (44.03)	518 (45.56)
Hospital	493 (12.98)	280 (10.52)	213 (18.73)
Clinic and others	432 (11.38)	302 (11.34)	130 (11.44)
Comorbidity, n (%)			
Interstitial pulmonary disease	1,303 (34.30)	930 (34.94)	373 (32.81)
COPD	272 (7.16)	176 (6.61)	96 (8.44)
Other respiratory disease	34 (0.89)	22 (0.83)	12 (1.06)
Diabetes	592 (15.58)	370 (13.90)	222 (19.53)
Mild liver disease	568 (14.95)	386 (14.50)	182 (16.01)
Moderate or severe liver disease	19 (0.50)	16 (0.60)	3 (0.26)
Renal failure	70 (1.84)	41 (1.54)	29 (2.55)
Chronic kidney disease	12 (0.32)	4 (0.15)	8 (0.70)
CCI, mean (SD)	1.33 (1.64)	1.26 (1.56)	1.49 (1.78)
CCI, n (%)			
0	1,497 (39.41)	1,072 (40.27)	425 (37.38)
1	1,048 (27.59)	759 (28.51)	289 (25.42)
2	570 (15.00)	385 (14.46)	185 (16.27)
≥3	684 (18.00)	446 (16.75)	238 (20.93)

SD, standard deviation; PDC, proportion of days covered; INH, isoniazid; RIF, rifampin; EMB, ethambutol; PZA, pyrazinamide; COPD, chronic obstructive pulmonary disease; CCI, Charlson comorbidity index.

Table 2 shows the estimated OR for adherence determined using logistic regression analysis. Younger patients were more likely to adhere to treatment (age 20–39 years, OR = 3.40; 40–49 years, 2.43; 60–79 years, 2.14) than those older than 80 years. Females and patients with health insurance were more likely to adhere to treatment than males and patients without health insurance, respectively. CCI was not statistically significant.

**TABLE 2:** Factors associated with adherence to initial treatment.

	Odds ratio (95% CI)
Age at index date (vs. age ≥80 years)	
20‒39	**3.40 (2.51–4.59)**
40‒59	**2.43 (1.81–3.24)**
60‒79	**2.14 (1.61–2.85)**
Sex	
Male (vs. female)	**0.80 (0.69–0.93)**
Type of insurance	
Health insurance (vs. medical aid)	**1.77 (1.37–2.28)**
CCI (vs. CCI = 0)	
1	1.01 (0.81–1.25)
2	0.92 (0.69–1.22)
≥3	0.93 (0.66–1.30)
Type of provider (vs. tertiary hospital)	
General hospital	**0.71 (0.60–0.85)**
Hospital	**0.46 (0.36–0.58)**
Clinic and others	**0.74 (0.57–0.95)**
Comorbidity (vs. without comorbid condition)	
Interstitial pulmonary disease	**1.23 (1.01–1.50)**
COPD	0.99 (0.74–1.33)
Other respiratory disease	0.56 (0.27–1.18)
Diabetes	0.86 (0.68–1.10)
Mild liver disease	0.96 (0.77–1.21)
Moderate or severe liver disease	3.43 (0.93–12.6)
Renal failure	0.97 (0.54–1.73)
Chronic kidney disease	0.17 (0.04–0.68)

CCI, Charlson Comorbidity index; COPD, chronic obstructive pulmonary disease.

The bold values are statistically significant.

### Initial treatment outcomes

**FIGURE 1:**
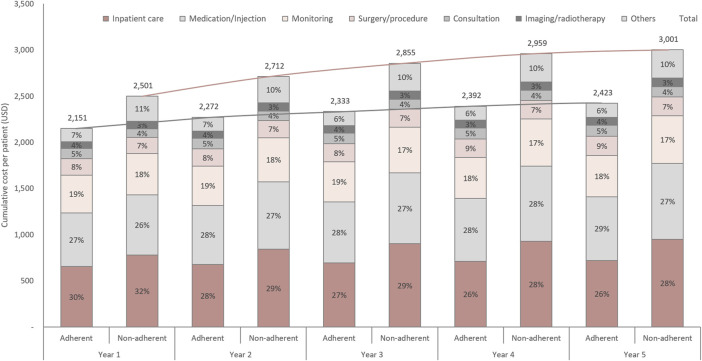
Initial treatment outcomes and retreatment rate in adherent and non-adherent groups. The height of the bars represents the proportion of patients. The initial number of adherent and non-adherent cells was 100%. MDR-TB, multidrug-resistant tuberculosis.

Figure 1 and Supplementary Table S1 show the treatment outcomes of DS-TB. After the initial TB treatment, 19.00% (*n* = 216) of patients in the non-adherent group and 5.63% (*n* = 150) of patients in the adherent group underwent retreatment. For initial treatment, the completion rate per 6 months was 0.68 (95% CI 0.66–0.71) in the adherent group and 0.49 (95% 0.45–0.54) in the non-adherent group, and the rate ratio was 1.37 (95% 1.25–1.51). Throughout the follow-up period, 1.69% of adherent and 1.14% of non-adherent patients were switched to MDR treatment. However, the rate ratio was not significant (1.29, 95% CI 0.58–2.93). The cumulative incidences of initial treatment completion and switching to MDR treatment are shown in Supplementary Figure S2.

### Treatment cost

**FIGURE 2:**
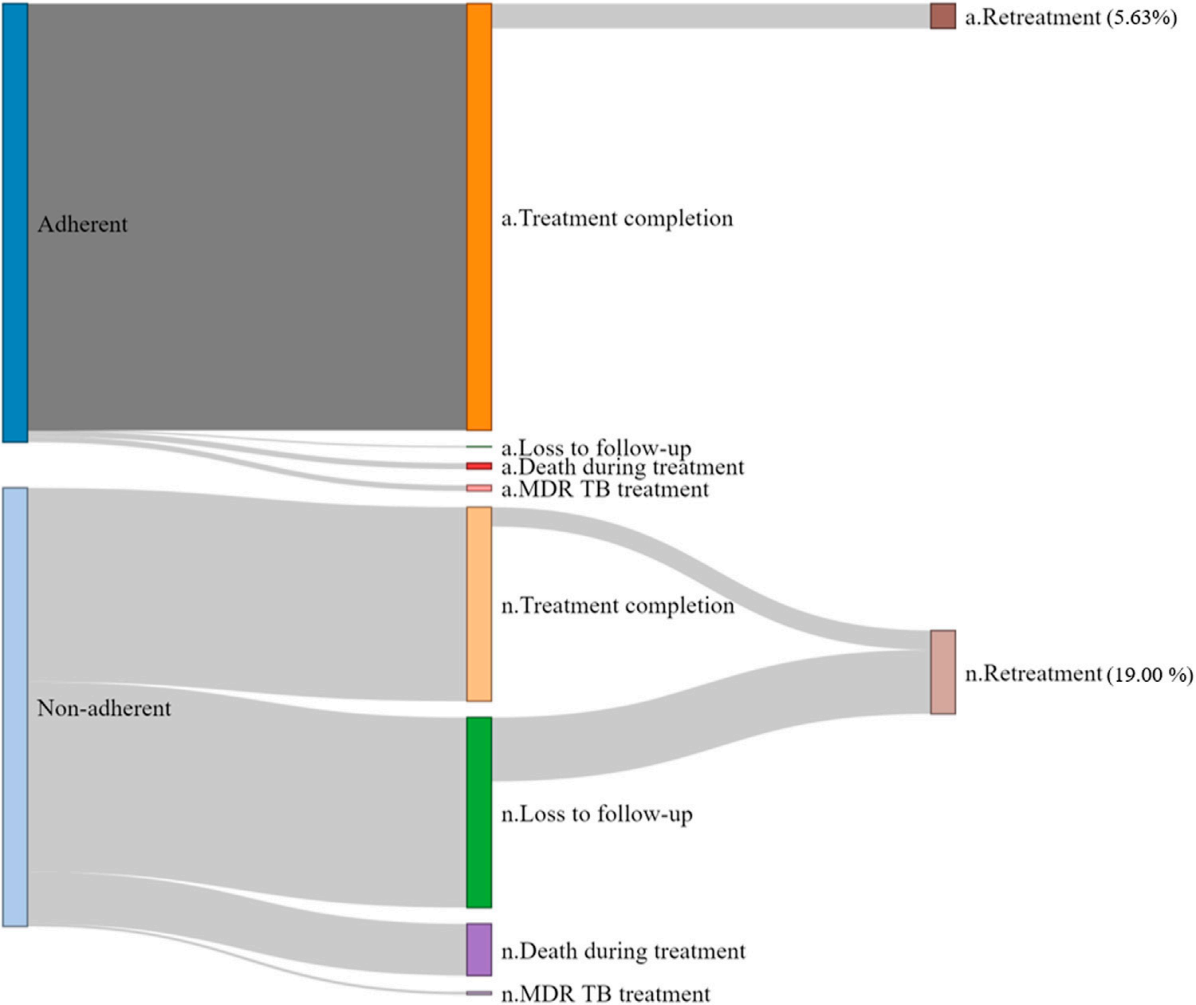
Tuberculosis-related cumulative cost per patient. Each line is a trend line for each group.

Figure 2 shows the cumulative TB-related costs adjusted for the censored data. Notably, the proportion of patients seeking inpatient care was higher in the non-adherent group than in the adherent group (32% vs. 30% in the first year; 28% vs. 26% in the fifth year). The proportion of medication costs was higher but the total cost was lower (USD 692 vs. USD 823 in the fifth year) in the adherent group than in the non-adherent group. The cumulative all-cause costs are shown in Supplementary Figure S3. In the first year, TB-related treatment costs accounted for 70.1% of all-cause costs in the adherent group and 76.1% in the non-adherent group, whereas in the fifth year, these values were 29.1 and 35.8%, respectively. The monthly cost per patient during the initial treatment, according to the treatment outcomes, is shown in Supplementary Figure S4. Patients who completed the initial treatment spent a mean of USD 208 per month. The monthly cost for deceased patients was highest (USD 2,395), followed by those who discontinued therapy (USD 498 per month). Generally, adherent patients incur lower costs compared to non-adherent patients, regardless of the treatment outcomes.

We estimated the relative ratio of monthly costs using a generalized linear model (Table 3). The monthly TB-related costs of the adherent group were 0.89-fold (95% CI 0.82–0.98) higher than those of the non-adherent group, whereas all-cause cost was not associated with adherence (relative ratio = 0.94, 95% CI 0.86–1.02). Additionally, TB-related costs borne by males were 1.21-fold (1.14–1.29) higher than that borne by females. Among the other factors examined, comorbidities, such as chronic obstructive pulmonary disease, diabetes, and renal failure, were also associated with both all-cause and TB-related monthly costs.

**TABLE 3:** Factors associated with the monthly cost per patient.

	Relative ratio (95% CI)	
	Tuberculosis-related cost	Total cost
Adherence (vs. non-adherent, PDC <0.8)		
Adherent (PDC ≥0.8)	**0.89 (0.82–0.98)**	0.94 (0.86–1.02)
Treatment outcomes (vs. treatment completion)		
Loss to follow-up from treatment	**2.52 (2.24–2.83)**	**2.48 (2.23–2.76)**
Death during treatment	**6.30 (5.34–7.42)**	**5.24 (4.51–6.09)**
Initiation of MDR-TB treatment	**5.67 (4.14–7.77)**	**4.64 (3.48–6.19)**
Age at index date (vs. age ≥80 years)		
20‒39	**0.53 (0.46–0.60)**	**0.47 (0.41–0.53)**
40‒59	**0.61 (0.53–0.69)**	**0.62 (0.55–0.70)**
60‒79	**0.82 (0.72–0.94)**	**0.84 (0.75–0.95)**
Sex		
Male (vs. female)	**1.21 (1.14–1.29)**	**1.15 (1.09–1.22)**
Type of insurance		
Health insurance (vs. medical aid)	**0.88 (0.78–0.98)**	**0.77 (0.69–0.85)**
CCI (vs. CCI = 0)		
1	**1.17 (1.06–1.28)**	**1.11 (1.02–1.21)**
2	1.08 (0.96–1.22)	**1.15 (1.03–1.29)**
≥3	**1.41 (1.22–1.63)**	**1.48 (1.29–1.69)**
Type of index provider (vs. tertiary hospital)		
General hospital	0.92 (0.86–0.99)	**0.93 (0.87–0.99)**
Hospital	0.92 (0.83–1.01)	0.94 (0.85–1.03)
Clinic and others	0.27 (0.24–0.30)	**0.37 (0.33–0.40)**
Comorbidity (vs. without comorbid condition)		
Interstitial pulmonary disease	**0.87 (0.80–0.94)**	**0.86 (0.79–0.93)**
COPD	**1.17 (1.03–1.33)**	**1.10 (0.98–1.23)**
Other respiratory disease	0.87 (0.63–1.20)	0.94 (0.70–1.27)
Diabetes	**1.12 (1.01–1.24)**	**1.11 (1.01–1.22)**
Mild liver disease	1.06 (0.97–1.17)	1.05 (0.96–1.14)
Moderate or severe liver disease	0.65 (0.42–0.99)	0.79 (0.53–1.17)
Renal failure	**2.47 (1.92–3.18)**	**2.71 (2.15–3.41)**
Chronic kidney disease	1.46 (0.81–2.61)	1.33 (0.77–2.28)

PDC, proportion of days covered; CCI, Charlson comorbidity index; COPD, chronic obstructive pulmonary disease.

The bold values are statistically significant.

## Discussion

Using representative real-world data, we observed higher treatment failure, retreatment rates, and economic burden in non-adherent patients with DS-TB than in adherent patients with DS-TB. Many studies showed that interventions, such as directly observed therapy, financial incentives, education, and national public-private mix projects to increase adherence, significantly improve treatment outcomes in patients with TB (WHO, 2003; Lei et al., 2015; Alipanah et al., 2018; Müller et al., 2018; Kang et al., 2021). However, only a few studies reported the relationship between treatment costs and TB adherence. In addition to studying this association, we evaluated cost drivers, which are often incompletely reported (Diel et al., 2014). Our results suggest that adherence is a critical factor associated with cost-effective TB management, which lowers TB treatment costs and improves treatment outcomes. As each country has a unique medical care and cost system, we used a generalized linear model to present the relative ratio of monthly costs according to adherence and observed that the adherent group had significantly lower treatment costs compared to the non-adherent group (relative ratio: 0.89, 95% CI 0.82–0.98). Adherence contributed to TB-related costs rather than to total costs. Cost breakdown analysis revealed that the proportion of inpatient care costs was higher in the non-adherent group, whereas medication costs were higher in the adherent group.

Previous studies did not clearly indicate an association between adherence and economic outcomes (Diniawati and Wibowo, 2018; Chimeh et al., 2020). One study suggested that good adherence results in lower medical costs, with the medical cost for patients showing an outcome of treatment completion was estimated to be 17% higher than the cost estimate of the national guideline-based treatment in South Africa. The results suggested that adherence leads to reduced TB treatment costs; however, actual adherence was not evaluated, and the comparator was a hypothetical cost described in national treatment guidelines. In addition, the results were based on a small pediatric sample evaluated for a short period (Budgell et al., 2018). Lei *et al.* reported an association between adherence and treatment costs in 481 Chinese patients with TB. The risk of non-adherence was significantly higher (OR: 2.08, 95% CI: 1.35–3.19) in the group with monthly treatment costs of 450 renminbi or higher than in the group with monthly treatment costs of less than 450 renminbi, suggesting that the burden of medical costs affected treatment adherence (Lei et al., 2016). Both studies indirectly suggested that adherence leads to reduced TB treatment costs. Therefore, to the best of our knowledge, this is the first study to reveal TB treatment costs according to adherence with a representative population, actual expenses, and long-term follow-up period that correlates with an increase in adherence and a reduction in medical costs.

In addition to treatment cost data, the treatment outcomes of patients with TB according to adherence were determined, which showed that the treatment failure rate was higher in the non-adherent group than in the adherent group. Moreover, regardless of adherence, the treatment cost incurred by patients increases when treatment is unsuccessful, mainly because of death and the development of MDR-TB. We evaluated the proportion of patients who underwent retreatment, whereas a previous study (Chimeh et al., 2020) directly measured unsuccessful treatment outcomes, such as failure and death. Poor adherence has been recognized as a major cause of treatment failure and drug resistance (Bell and Yach, 1988; Yach, 1988). In this study, the retreatment rate in the non-adherent group was more than three-fold higher than that in the adherent group. A previous cost-effectiveness study predicted an increase in the number of new TB cases and TB-related deaths and costs with respect to non-adherence to therapy (Patel et al., 2017). In addition to death and treatment failure, the higher retreatment rate in the non-adherent group in our study may explain the significant increase in cumulative cost by year. In addition, we found that the non-adherent group spent more inpatient care, which was previously reported (Gullón-Blanco et al., 2016) as the major cost driver. Therefore, TB management to improve patient adherence may reduce the national budget for TB control. Although public-private mix projects and directly observed therapy require large financial investments, they improve clinical outcomes in patients with TB and lead to lower treatment costs. Considering the social impact of TB and cost of preventing infections due to the successful treatment of TB, the continuous implementation of policies to improve adherence to TB treatment should be supported, and cost-effectiveness studies considering all related costs should be conducted. However, poor TB management and control may lead to poor outcomes, particularly during situations requiring reduced access to medical facilities, such as during the coronavirus disease 2019 pandemic.

Comorbidities, such as diabetes and renal failure, were not directly associated with patient adherence but were associated with TB-related monthly costs. Adherence and comorbidity were not related; however, many factors have been shown to be associated with adherence to TB treatment, including patient characteristics, treatment regimens, and healthcare settings (WHO, 2003). Previous systematic reviews and studies showed that diabetes and chronic renal failure lead to an increase in poor outcomes, particularly death, in patients with TB (Baker et al., 2011; Baghaei et al., 2014; Barreto-Duarte et al., 2021). Interestingly, interstitial pulmonary disease is associated with adherence and costs. Although on studies have reported the relationship between clinical and economic outcomes, interstitial pulmonary disease may be related to TB. Further studies should focus on treatment costs incurred by patients with TB and comorbidities.

This study had several limitations. Because we evaluated claims data, misclassification of exposure and covariates may have occurred. To reduce misclassification, we required at least two claims with 3 TB drugs within 1 month when defining patients with TB. Although smoking status, education level, and income might affect one’s adherence (Kardas et al., 2013), there was lacking information. However, we used types of insurance plan such as medical aid, which reflected the economic status of house income. As adherence was measured during the follow-up period, patients who died within 5 months were categorized as non-adherent. Hence, the death rate may have been overestimated, and the results should be interpreted with caution. However, retreatment rates may have been underestimated. However, the death rate after the initial treatment was still higher in the non-adherent group than in the adherent group, which is consistent with previously reported results (Chimeh et al., 2020).

## Conclusion

Adherence to TB treatment is associated with the clinical burden, and good adherence leads to a reduction of the economic burden on patients with DS-TB. Commitment programs to improve patients’ adherence can be helpful to manage TB nationwide by reducing cost and would contribute to the end TB strategy worldwide.

## Data Availability

The datasets presented in this article are not readily available because this study used the National Health Insurance Service-National Sample Cohort (NHIS-NSC) 2.0 database in South Korea. Data were obtained from the NHIS with permission. Requests to access the datasets should be directed to http://nhiss.nhis.or.kr/bd/ab/bdaba021eng.do.

## Abbreviations

CCI, Charlson comorbidity index; CI, confidence interval; DS-TB, drug-susceptible TB; ICD-10, International Statistical Classification of Diseases and Related Health Problems, 10th Revision; LTFU, loss to follow-up; MDR-TB, multidrug-resistant TB; NHIS, National Health Insurance Service; NHIS-NSC, National Health Insurance Service–National Sample Cohort; OR, odds ratio; PDC, proportion of days covered; TB, tuberculosis.
