# Supplementary material for: Real-world association of adherence with outcomes and economic burden in patients with tuberculosis from South Korea claims data
Source: Front Pharmacol. 2022 Aug 16;13:918344. doi: 10.3389/fphar.2022.918344 (PMC9424769; doi:10.3389/fphar.2022.918344)
Supplement: Supplementary file 1 [file DataSheet1.PDF]

Supplementary Online Content

Supplemental Tables

Table S1. Incidence of treatment outcomes

Supplemental Figures

Figure S1. Study design.

Figure S2. Cumulative incidence rate of initial treatment outcomes. A. Initial treatment completion, B. Initiation of multidrug-resistant treatment.

Figure S3. All-cause cumulative cost per patient.

Figure S4. Monthly cost per patient according to treatment outcome..

Table S1. Incidence of treatment outcomes

	All patients (n = 3,799)	Adherent group (n = 2,662)	Non-adherent group (n = 1,137)
Cumulative incidence,[a] n (%)			
Initial treatment completion	3,093 (81.42)	2,590 (97.30)	503 (44.24)
Retreatment after initial treatment	366 (9.63)	150 (5.63)	216 (19.00)
Initiation of MDR treatment			
During entire follow-up period	58 (1.53)	45 (1.69)	13 (1.14)
During initial treatment	41 (1.08)	34 (1.28)	7 (0.62)
All-cause death	493 (12.98)	222 (8.34)	271 (23.83)
During initial treatment	170 (4.47)	36 (1.35)	134 (11.79)
After initial treatment	323 (8.50)	186 (6.99)	137 (12.05)
Total person-year	2,408	1,900	508
Incidence per six months (95% CI)			
Initial treatment completion	0.64 (0.62–0.67)	0.68 (0.66–0.71)	0.49 (0.45–0.54)
Initiation of MDR-TB treatment	0.01 (0.01–0.01)	0.01 (0.01–0.01)	0.01 (0.00–0.01)
Time to event (year), median (IQR)			
Initial treatment completion	0.65 (0.512–0.811)	0.65 (0.51–0.80)	0.67 (0.53–0.89)
Initiation of MDR-TB treatment	0.22 (0.085–0.348)	0.22 (0.11–0.37)	0.13 (0.00–0.31)

MDR-TB, multidrug-resistant tuberculosis; IQR, interquartile range.
[a]Each outcome was interpreted individually. The sum of the percentages was not 100%.

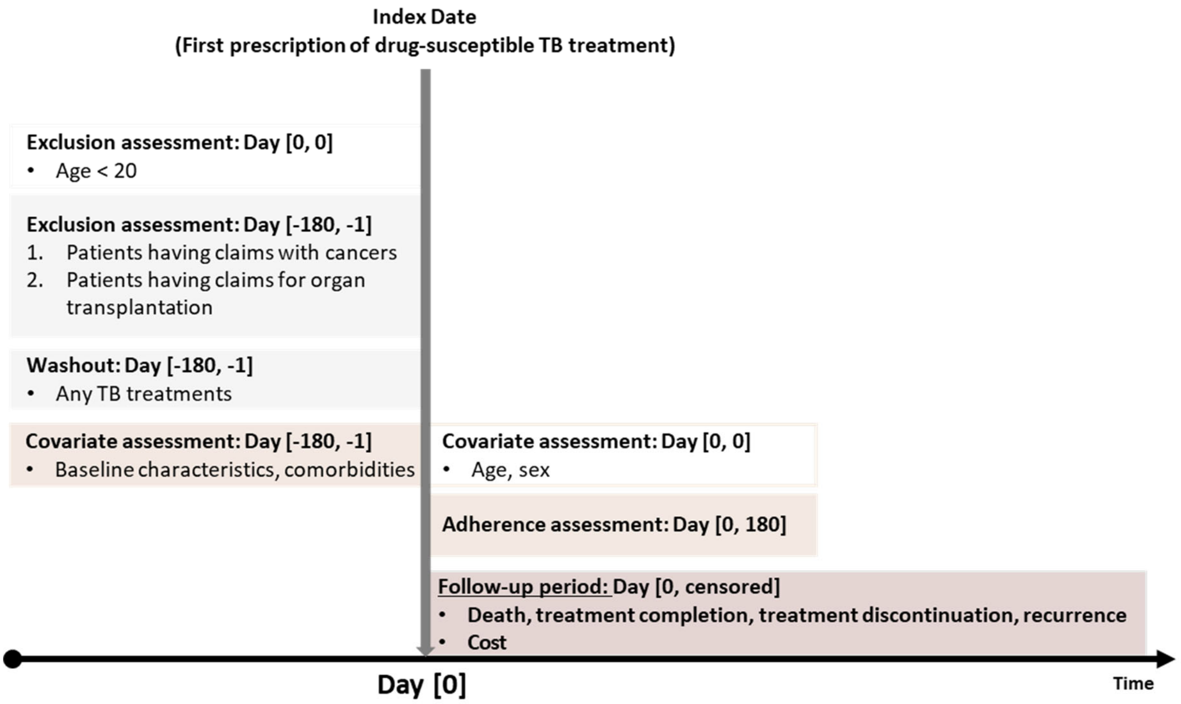

Figure S1. Study design.

TB, tuberculosis.

A. Initial treatment completion

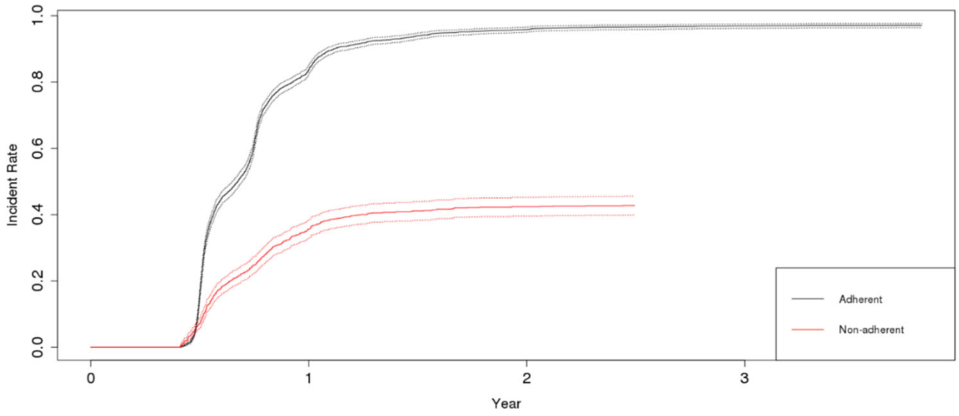

B. Initiation of multidrug-resistant treatments

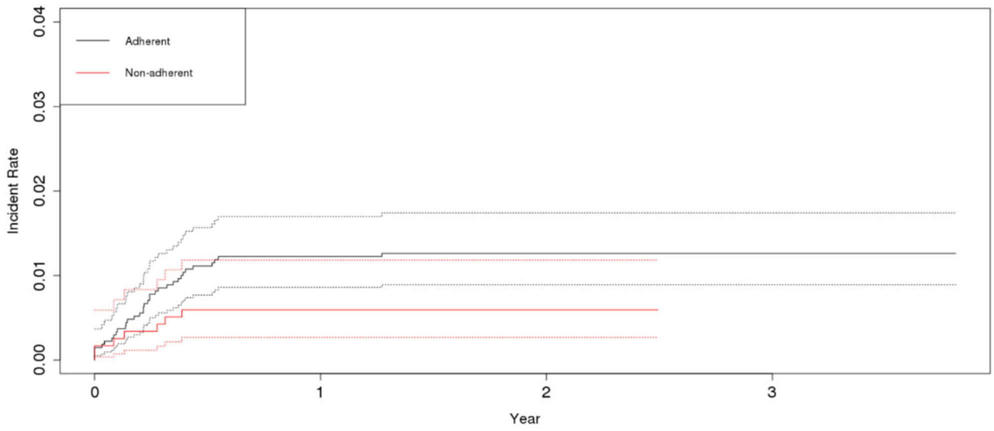

Figure S2. Cumulative incidence rate of initial treatment outcomes. A. Initial treatment completion, B. Initiation of multidrug-resistant treatment.

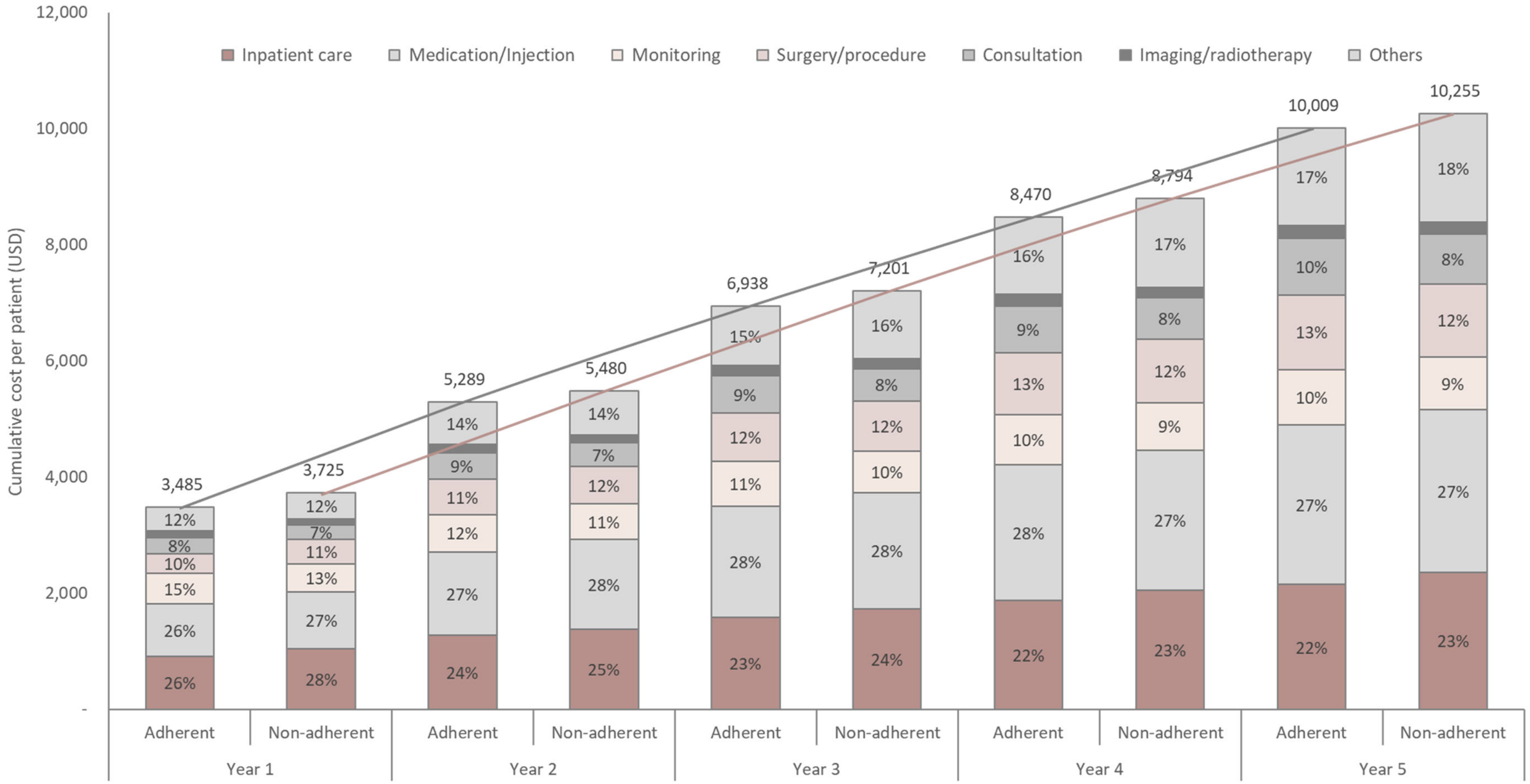

Figure S3. All-cause cumulative cost per patient.

Each line represents a trend line for each group.

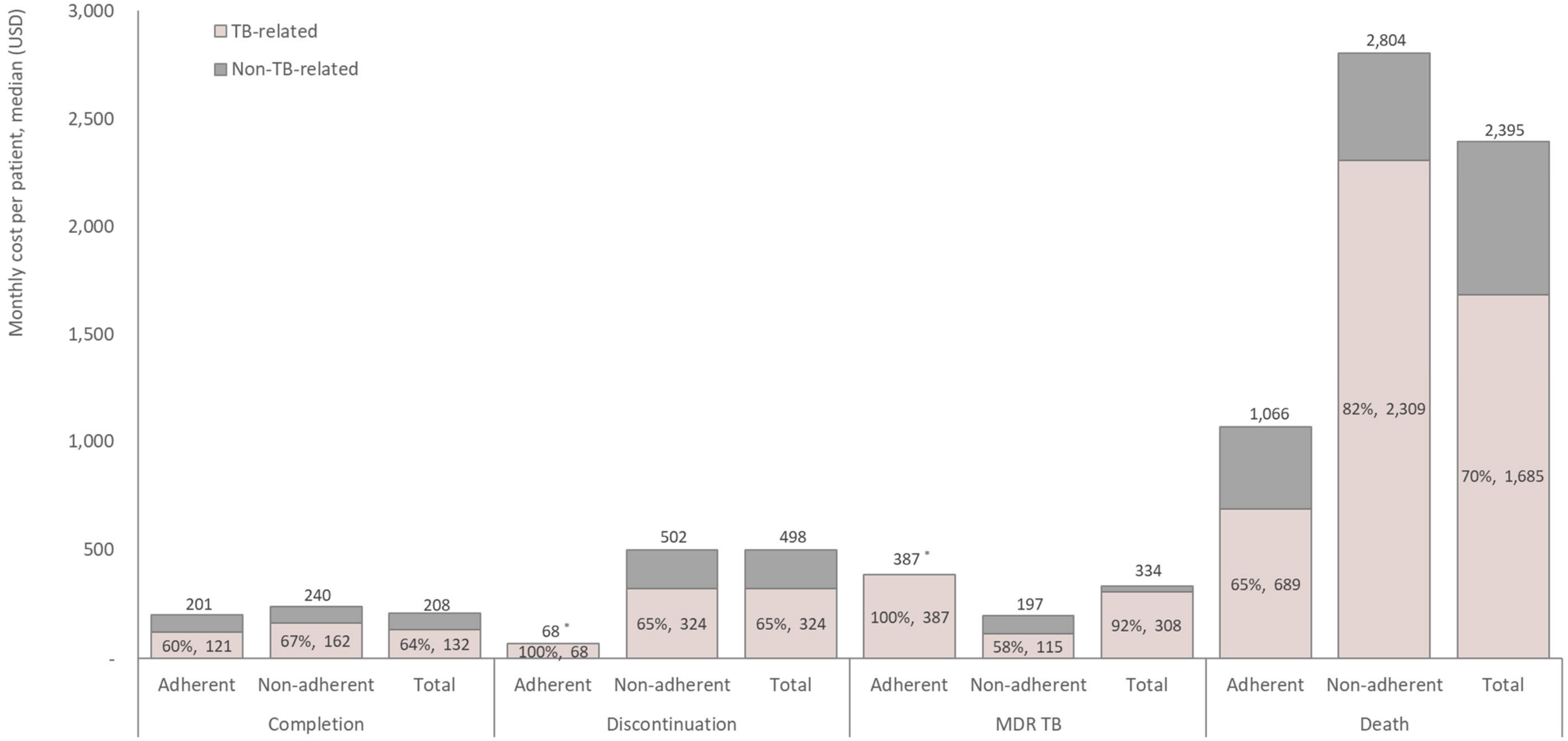

Figure S4. Monthly cost per patient according to treatment outcome.

TB, tuberculosis.

*All-cause cost was the same as TB-related cost.
